# Acceptance and Commitment Therapy delivered in the workplace – Effects of a 5-week training program on corporate employees’ psychological flexibility

**DOI:** 10.1192/j.eurpsy.2025.2005

**Published:** 2025-08-26

**Authors:** Á. K. Magyary, Z. Demetrovics, Á. Zsila, N. Z. Mészáros

**Affiliations:** 1 Doctoral School of Psychology; 2 Institute of Psychology, ELTE Eötvös Loránd University; 3 Institute of Psychology, Catholic University of Pázmány Péter, Budapest, Hungary; 4 Centre of Excellence in Responsible Gaming, University of Gibraltar, Gibraltar, Gibraltar; 5 College of Education, Psychology and Social Work, Flinders University, Adelaide, Australia; 6 Institute of Psychology, University of Pécs, Pécs, Hungary

## Abstract

**Introduction:**

A growing body of research has demonstrated the positive effects of Acceptance and Commitment Therapy (ACT) delivered in the workplace on employees’ well-being and psychological flexibility. Delivering ACT in the workplace aims to enhance employee performance and reduce stress levels by promoting psychological flexibility, a skill to consciously live in the present, observing thoughts and emotions, and engaging in value-driven actions.

**Objectives:**

The short-term effects of a 5-week ACT-based psychological flexibility training program were tested.

**Methods:**

Participants were 21 corporate employees (62 % women, M*age* = 43.9 years, SD = 9.5) recruited from a large company in the financial sector. The six core processes of psychological flexibility were examined before the first training session and after the fifth training session of the 5-week training. Psychological flexibility, value-driven living, thought suppression, five facets of mindfulness and cognitive fusion were assessed.

**Results:**

Results showed a statistically significant difference in one core process of psychological flexibility, namely *Contact with the present moment.* *Acting with awareness*, *Describing* and *Nonjudge* showed significant difference before and after the training. Presumably, changes in *Cognitive defusion* and *Non-reactivity* are also evident, however results did not show significant difference.

**Conclusions:**

The present research has provided further empirical evidence for the effectiveness of ACT-based psychological flexibility training with regard to three aspects of *Contact with the present moment*, which is a core process of psychological flexibility (*Acting with awareness, Describing,* and *Nonjudge*). The findings provide a basis for future research to investigate longer-term effects, including monitoring how participants in the research program practice the learnt techniques in their everyday lives.

**Disclosure of Interest:**

Á. Magyary Grant / Research support from: ÁM was supported by the ÚNKP-23-2 New National Excellence Program of the Ministry for Culture and Innovation from the source of the National Research, Development and Innovation Fund., Z. Demetrovics: None Declared, Á. Zsila: None Declared, N. Mészáros: None Declared

